# Diffusion tensor imaging tractography of the optic radiation for epilepsy surgical planning: A comparison of two methods

**DOI:** 10.1016/j.eplepsyres.2011.07.019

**Published:** 2011-11

**Authors:** Gavin P. Winston, Laura Mancini, Jason Stretton, Jonathan Ashmore, Mark R. Symms, John S. Duncan, Tarek A. Yousry

**Affiliations:** a Epilepsy Society MRI Unit, Departmental of Clinical and Experimental Epilepsy, UCL Institute of Neurology, Queen Square, London, WC1N 3BG, England, UK; b Lysholm Department of Neuroradiology, National Hospital for Neurology and Neurosurgery, Queen Square, London, WC1N 3BG, England, UK; c Academic Neuroradiological Unit, Department of Brain Repair and Rehabilitation, UCL Institute of Neurology, Queen Square, London, WC1N 3BG, England, UK

**Keywords:** Diffusion tensor imaging, Epilepsy surgery, Optic radiation, Tumours, Visual field deficits

## Abstract

The optic radiation is a key white matter structure at risk during epilepsy surgery involving the temporal, parietal or occipital lobes. It shows considerable anatomical variability, cannot be delineated on clinical MRI sequences and damage may cause a disabling visual field deficit. Diffusion tensor imaging tractography allows non-invasive mapping of this pathway. Numerous methods have been published but direct comparison is difficult as patient, acquisition and analysis parameters differ.

Two methods for delineating the optic radiation were applied to 6 healthy controls and 4 patients with epileptogenic lesions near the optic radiation. By comparing methods with the same datasets, many of the parameters could be controlled. The first method was previously developed to accurately identify Meyer's loop for planning anterior temporal lobe resection. The second aimed to address limitations of this method by using a more automated technique to reduce operator time and to depict the entire optic radiation.

Whilst the core of the tract was common to both methods, there was significant variability between the methods. Method 1 gave a more consistent depiction of Meyer's loop with fewer spurious tracts. Method 2 gave a better depiction of the entire optic radiation, particularly in more posterior portions, but did not identify Meyer's loop in one patient.

These results show that whilst tractography is a promising technique, there is significant variability depending on the method chosen even when the majority of parameters are fixed. Different methods may need to be chosen for surgical planning depending on the individual clinical situation.

## Introduction

The optic radiation is a key white matter structure conveying visual information from the lateral geniculate nucleus (LGN) to the primary visual cortex in the occipital lobe. Fibres from the inferior retina, representing the superior visual field, pass anteriorly from the LGN into the temporal lobe looping around the temporal horn of the lateral ventricle (Meyer's loop) before passing posteriorly (Meyer, 1907). Fibres from the superior retina, representing the inferior visual field, pass directly posteriorly through the parietal lobe to the occipital lobe. The extensive course involving temporal, parietal and occipital lobes means that this structure is at risk during neurosurgery with damage leading to a visual field deficit.

In the 30% of patients with focal epilepsy who fail to respond to drug therapy, surgical treatment may be considered. Anterior temporal lobe resection, the most common operation, is highly effective (Wiebe et al., 2001) but damage to Meyer's loop can cause a superior quadrantanopia. Between 48% (Nilsson et al., 2004) and 100% (Barton et al., 2005) of patients develop a visual field deficit and a quarter fail to meet UK criteria for driving (Manji and Plant, 2000). Epileptogenic lesions may however lie at any point along the course of the optic radiation. Neurosurgery can thus lead to a variety of visual field deficits and the potential benefits of the neurosurgery must be balanced against the risk to vision.

Conventional clinical MRI sequences cannot delineate the optic radiation, which shows considerable anatomical variability (Ebeling and Reulen, 1988). Diffusion tensor imaging measures the directional diffusion of water in tissues (Basser, 1995) and tractography algorithms can be applied to these data to non-invasively map out white matter pathways, including the optic radiation (Mori and van Zijl, 2002). Tractography is a complex and time-consuming process with results that depend upon the method employed. It is difficult to directly compare published methods as there are many differences in the patient population, scan acquisition (e.g. scanner manufacturer, field strength, voxel size, number of gradient directions, b-value) and analysis parameters (e.g. software product, type of tractography algorithm, seed region and waypoint location, stopping criteria).

The aim of the present study was to compare two methods for delineating the optic radiation using the same datasets and controlling as many parameters as possible. We have an optimised DTI acquisition sequence used in longitudinal studies and therefore wished to compare different tractography analysis methods to determine the level of agreement between them and to identify the method that more reliably identifies different components of the optic radiation for use in different types of epilepsy surgery.

The first method was designed for planning anterior temporal lobe resections and pays special attention to accurate identification of Meyer's loop. This method has been previously published (Yogarajah et al., 2009) and reliability, strengths and weaknesses established. Tractography results obtained correlate with effect of anterior temporal lobe resection on visual fields and the method is reproducible. However, it is time consuming for the operator and since it concentrates on Meyer's loop, subjective experience suggests that it performs less well with more posterior parts of the optic radiation. Thus a second method was designed to semi-automatically identify the location of the LGN thus reducing operator time and to use this region to delineate the whole of the optic radiation.

## Methods

### Subjects

We studied 6 healthy controls (age 25–53 years, 4 males) and 4 patients with medically refractory epilepsy (age 18–42 years, 2 males) undergoing pre-surgical evaluation at the National Hospital for Neurology and Neurosurgery, London, UK. The study was approved by the National Hospital for Neurology and Neurosurgery and the Institute of Neurology Joint Ethics Committee, and informed written consent was obtained from all subjects. Patient demographics and imaging diagnoses are listed in Table 1.

### Magnetic resonance data

MRI studies were performed on a 3T GE Excite II scanner (General Electric, Waukesha, Milwaukee, WI, USA). Standard imaging gradients with a maximum strength of 40 mT m^−1^ and slew rate 150 T m^−1^ s^−1^ were used. All data were acquired using a body coil for transmission, and an eight channel phased array coil for reception. DTI data were acquired using a cardiac-triggered single-shot spin-echo echo planar imaging (EPI) sequence (Wheeler-Kingshott et al., 2002) with TE = 73 ms. Sets of 60 contiguous 2.4-mm thick axial slices were obtained, covering the whole brain, with diffusion sensitizing gradients applied in each of 52 non-collinear directions [*b* value 1200 mm^2^ s^−1^, *δ* = 21 ms, *Δ* = 29 ms, using full gradient strength of 40 mT m^−1^)] along with six non-diffusion weighted scans. The gradient directions were calculated and ordered as described elsewhere (Cook et al., 2007). The field of view was 24 cm with an acquisition matrix of 96 × 96 giving isotropic 2.4 mm resolution. The matrix was zero filled to 128 × 128 during reconstruction giving a reconstructed voxel size of 1.875 mm × 1.875 mm × 2.4 mm. The DTI acquisition time for a total of 3480 image slices was approximately 25 min (depending on subject heart rate).

### DTI preprocessing

The scans were transferred to a Linux-based Sun Ultra workstation in DICOM format and converted to a single multivolume Analyze 7.5 format file using locally written software. Images were viewed in a movie loop to ensure that no subjects with significant movement were included. Eddy current correction of the DTI data was performed using the eddycorrect tool in FSL v4.0.1 (Smith et al., 2004).

A multi-tensor model was fitted to the eddy corrected diffusion data using the Camino toolkit version 2 release 767 (Cook et al., 2006). Voxels in which a single tensor fitted the data poorly were identified using a spherical-harmonic voxel-classification algorithm (Alexander et al., 2002). In these voxels a two tensor model was fitted, with the principal diffusion directions of the two diffusion tensors providing estimates of the orientations of the crossing fibres. In all other voxels, a single tensor model was fitted.

### Tractography

Tractography analysis was carried out using the Probabilistic Index of Connectivity (PICo) algorithm (Parker et al., 2003) implemented in Camino extended to deal with multiple fibres (Cook et al., 2004; Parker and Alexander, 2003) and a total of 50,000 iterations to reduce variability. An angular threshold of 180 degrees and a fractional anisotropy threshold of 0.1 were used in order to ensure that the paths detected would not erroneously enter areas of cerebrospinal fluid, and yet had sufficient angular flexibility to allow tracking of Meyer's loop.

The seed region, waypoint, endpoint and exclusion masks were the only factors differing between the methods and are described below. Connectivity distributions were generated from each voxel in the seed region and combined into an overall connectivity map representing the maximum observed connection probability to each voxel within the brain from all the voxels within the seed region. For display on fractional anisotropy maps, the connectivity map was thresholded to include only voxels with a connection probability greater than 5%, representing a compromise between retaining anatomically valid tracts, and removing obviously artefactual connections.

#### Method 1

**Figure 1:**
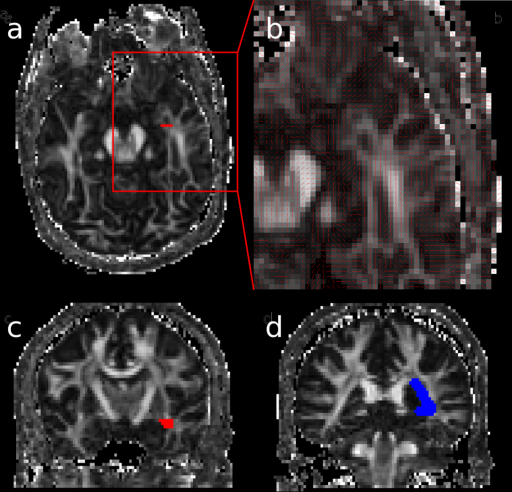
*Method 1*. Seed region in Meyer's loop near the LGN (red) in the axial (a) and coronal planes (c), with a close up of Meyer's loop (b). Waypoint in the lateral wall of the lateral ventricle (blue) identified in the coronal plane (d).

Fractional anisotropy and principal diffusion direction maps were used to identify the LGN by selecting the axial slice at the level of the transition from the posterior limb of the internal capsule to the cerebral peduncle. Voxels antero-lateral to the LGN across the base of Meyer's loop, with principal eigenvectors orientated in an antero-medial to postero-lateral orientation, were identified and used to define a seed region in a coronal plane (Fig. 1a–c). Contiguous voxels, with principal directions in an anterior–posterior direction, were also selected in order to cover the entire coronal cross-section of Meyer's loop, using a standardised seed region volume of 15 voxels (127 mm^3^).

In order to restrict the pathway to anatomically valid tracts, a waypoint was defined in the lateral wall of the occipital horn of the lateral ventricle at the posterior extent of the corpus callosum (Fig. 1d). Two exclusion masks were applied. Firstly, a whole brain midline exclusion mask and then a coronal exclusion mask to remove artefactual connections to adjacent white matter tracts, such as the fronto-occipital fasciculus, anterior commissure and uncinate fasciculus. An objective, iterative process was performed to determine the optimum location for this mask whereby the exclusion mask was moved posteriorly until it began to coincide with Meyer's loop, identified by a visible thinning of the estimated trajectory of the optic radiation, typically associated with a reduction in tract volume greater than 10% (Yogarajah et al., 2009).

#### Method 2

**Figure 2:**
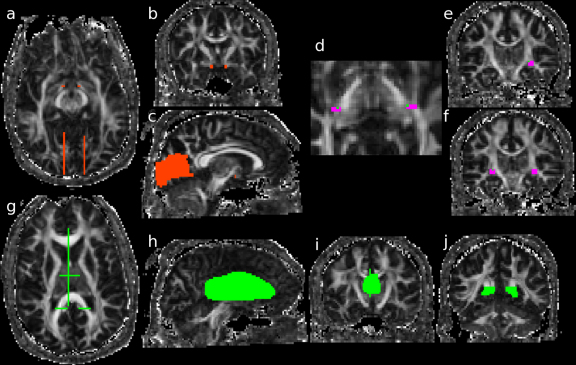
*Method 2*. Seed regions in the optic tract (red) in the axial (a) and coronal planes (b). Large regions of interest (ROIs) including voxels above and below the calcarine fissure (red) lateral to the midline in the axial (a) and sagittal planes (c), used both as seed region for the identification of the LGN region and as an endpoint for the final identification of the optic radiation. Seed regions in the LGN region (pink) in axial (d) and coronal planes (e, and f), with the voxel (green) closest to the LGN where the optic tract and optic radiation overlap. Exclusion masks (green) in the midline encompassing both the thalamus and corpus callosum (h), in the thalamus in a coronal plane (i), at the level of the trigone (j) and all three shown in the axial plane (g).

An automated method was used to identify the location of the LGN. This method consisted of first initiating tracts (with 5000 iterations) separately from two seed regions. For the first seed region, the optic tract between the chiasm and the LGN was identified on an axial slice. A coronal slice intersecting the optic tract was chosen on which 8 voxels were selected in the region of the optic tract (Fig. 2a and b). For the second seed region, the calcarine fissure was identified on a sagittal slice close to the midline. The most lateral slice on which the calcarine fissure was still visible was chosen on which a large area was identified including voxels above and below the calcarine fissure (Fig. 2a and c). No endpoints were defined on the tractography initiated separately from these two seed regions. The two tracts obtained from these two seed regions were then thresholded so that only connection probabilities higher than 0.2% were selected in order to eliminate some artefactual tracts whilst retaining fascicles reaching the LGN area posterolateral to the cerebral peduncle. The product of these two connectivity distributions gives a probability distribution for the location of the LGN with the maximum voxel of the distribution representing the closest region to the LGN where both optic tract and optic radiation overlap.

A second step of the analysis involved selecting a rectangular seed region comprising 12 voxels on each of two consecutive coronal slices including the area close to the LGN location identified with the automated method described and extending inferiorly from this location, in regions of high fractional anisotropy (Fig. 2d–f). The optic radiation was identified by seeding from this region using the same endpoint located above and below the calcarine fissure described above (Fig. 2c). Exclusion masks were also applied to each hemisphere positioned at the posterior region of the trigone, in the midline encompassing both the thalamus and corpus callosum and on a coronal slice in the medial thalamus, to eliminate erroneous tracts (Fig. 2g–j).

### Volume of optic radiation and degree of overlap

For each of the 20 hemispheres, the number of voxels in the tract (thresholded at 0.05) was calculated for the two methods individually, along with the number of voxels common to both methods. The Dice Similarity Score quantified the degree of overlap giving a figure between 0 (no overlap) to 1 (complete overlap):

$$\text{Dice Similarity Score}=2\times \frac{\text{numbers of voxels common to both methods}}{\left[\text{number of voxels with method}\;1+\text{number of voxels with method}\;2\right]}$$

### Measurements of Meyer's loop

The location of the temporal pole was identified in each hemisphere using the fractional anisotropy image (FA). The anterior extent of Meyer's loop from the tractography was then located to calculate the antero-posterior distance between the temporal pole and Meyer's loop (TP-ML distance) in voxels. These measurements were performed for each of the two methods. As the reproducibility of method 2 for identification of the TP-ML distance has not been previously assessed, the test–retest and inter-rater reliability of this distance was assessed using the intraclass correlation coefficient (SPSS Statistics 17.0).

## Results

### Visual comparison

**Figure 3:**
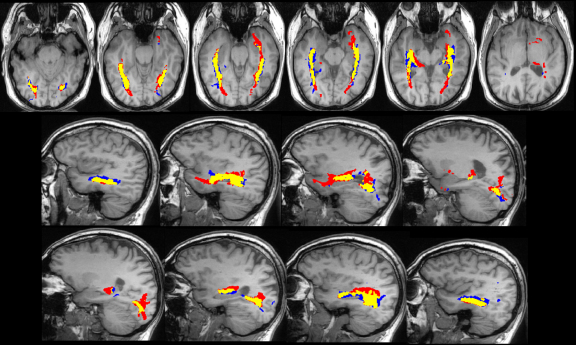
Optic radiation identified by Method 1 only (blue), Method 2 only (red) and both methods (yellow) in a control subject, overlaid on the T1-weighted image. The images are shown in radiological convention with axial slices from inferior to superior and sagittal slices from left to right.

**Fig. 4:**
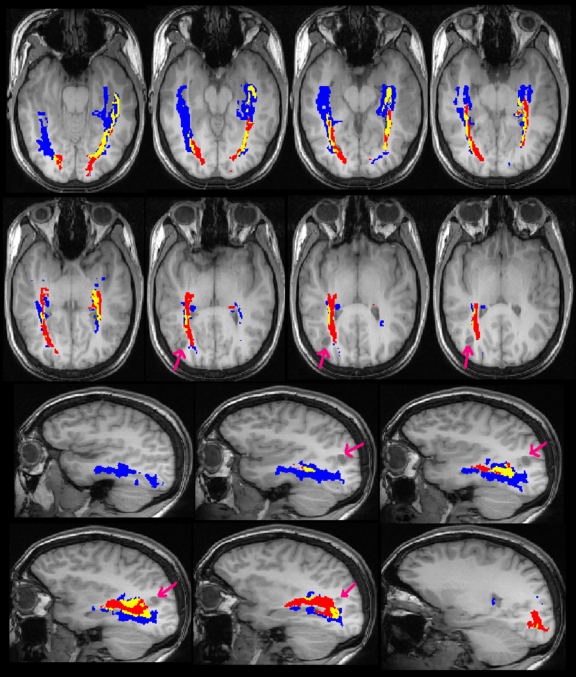
Optic radiation identified in patient 1 by Method 1 only (blue), Method 2 only (red) and both methods (yellow), overlaid on the T1-weighted image. The arrows indicate the right inferior parietal DNET. Image conventions as Fig. 3.

**Fig. 5:**
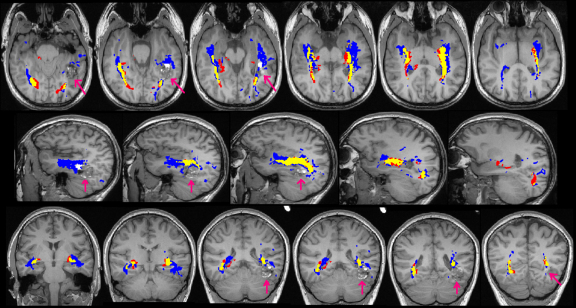
Optic radiation identified in patient 2 by Method 1 only (blue), Method 2 only (red) and both methods (yellow) overlaid on the T1-weighted image. The arrows indicate the left temporal cavernoma. Image conventions as Fig. 3, with the coronal images being from anterior to posterior.

Visual comparison of the results showed that the core of the delineated tract was common between the two methods both in healthy controls (Fig. 3) and patients with epilepsy (Figs. 4 and 5). Method 1, which was designed to depict Meyer's loop, gave a much clearer visualisation of this structure and the observer found it easier to perform measurements using this method. The frontal exclusion mask ensured fewer false tracts in the temporal pole and towards the anterior commissure (Fig. 3), which were commonly seen using Method 2, but it could not always eliminate the spurious tracts present in the insula (Fig. 5).

Method 2 however gave a better depiction of the optic radiation as it reached the visual cortex, with the paths from method 1 tending to thin out more posteriorly. In addition, method 2 tended to delineate the optic radiation more superiorly than method 1 which is important when considering the relationship between the optic radiation and lesions such as the inferior parietal DNET in Patient 1 (Fig. 4). Finally, method 2 failed to correctly delineate the portion of the optic radiation anterior to the large cavernoma in patient 2 (Fig. 5).

### Volume of optic radiation and degree of overlap

In controls, the average volume of the optic radiation was the same using both methods (Table 2). However, in patients there was a trend to lower tract volumes using method 2 (two-tailed paired *t*-test *p* = 0.07) particularly in patient 2 ipsilateral to the lesion where the tractography failed.

The Dice Similarity Score revealed overlap varied between 0.160 and 0.612 (mean 0.400, standard deviation 0.132). Overlap was lower in the patients with epilepsy than in healthy controls (0.318 vs 0.456, two-tailed *t*-test *p* = 0.018). There was no significant change when the false tracts obtained in method 2 were masked out using the same frontal exclusion mask as in method 1.

### Measurements of Meyer's loop

Method 1 was successful in all subjects whilst method 2 failed to identify Meyer's loop on the side of pathology in patient 2. There was no significant difference between the mean ML-TP distance in either controls or patients (Table 2), although there was a trend towards a greater distance in patients (paired *t*-test, *p* = 0.148). For method 2, the intra-rater intraclass correlation coefficient was 0.826 with better agreement in controls than patients (0.898 vs 0.451). The measurements were identical in 13 hemispheres whilst in the other 6 hemispheres, there was up to 3 voxels difference in the TP-ML distance. With the second observer, method 2 did not produce an anatomically plausible tract in a single control hemisphere. Excluding this outlier, the inter-rater intraclass correlation coefficient was 0.560 (0.729 in controls and 0.319 in patients). There was agreement in 10 hemispheres and in the majority of the remainder the difference was 1 or 2 voxels. However there was a difference of 6 voxels in a single hemisphere of a patient.

## Discussion

Tractography is a powerful technique enabling non-invasive *in vivo* delineation of white matter tracts. A key assumption is that the results are a faithful representation of the underlying axonal microstructure and thus the white matter tracts themselves. It is not usually possible to compare the results to the gold standard of post mortem dissection. As tractography results are of great utility in planning neurosurgical procedures where the optic radiation lies near the lesion (Romano et al., 2009), it is important to have confidence in the results.

Tractography is subject to a number of limitations and potential errors. Data acquisition using echo-planar imaging may be subject to errors through motion, eddy currents, susceptibility artefacts and noise. The voxel size is several orders of magnitude greater than the underlying axonal structure, so voxels may contain multiple fibres or be subject to partial volume effects. Particular problems include kissing or crossing fibres and areas of high curvature, such as Meyer's loop. The model chosen to represent the underlying axonal structure and the tractography algorithm both make assumptions, such as the presence of a single fibre population in each voxel, which may not be valid.

The degree to which these potential sources of error affect the results depends on many variables chosen during the process making it difficult to compare the different methods of published studies. By controlling as many variables as possible, including scanner type, acquisition parameters, preprocessing and diffusion model, a more direct comparison between two tractography methods was possible.

Deterministic methods are often favoured as they provide visually attractive results, with only limited processing required. Tractography between the LGN and the occipital cortex has been used to depict the optic radiation (Catani et al., 2003), including the different layers (Yamamoto et al., 2005). Critically however these algorithms underestimate the anterior extent of Meyer's loop and this is not improved by increasing the number of gradient directions (Yamamoto et al., 2007). In 18 subjects, the TP-ML distance using a deterministic technique was 32–51 mm (mean 41 mm) compared to 17–42 mm (mean 30 mm) using a probabilistic technique (Nilsson et al., 2010). The latter is more concordant with dissectional data showing a TP-ML distance of 22–37 mm (mean 27 mm) (Ebeling and Reulen, 1988). A more recent study suggesting reconstruction of the entire human visual pathway conceded that Meyer's loop was only fully reconstructed in half of the healthy controls (Hofer et al., 2010). Despite these limitations, such algorithms have been applied during neurosurgery (Chen et al., 2009).

Probabilistic tractography is more time consuming both computationally and for the operator but better delineates Meyer's loop (Nilsson et al., 2010). Seed regions either lateral to the LGN to avoid the brachium of the superior colliculus (Behrens et al., 2003) or within the apex of Meyer's loop (Powell et al., 2005; Yogarajah et al., 2009) have been used. Both deterministic and probabilistic tractography results are sensitive to the location of the seed region. Even a single voxel displacement of seed region leads to a coefficient of variation of 2.5% in the mean FA of the optic radiation (Ciccarelli et al., 2003). Such variability is confirmed by the relatively low Dice Similarity Scores in this study, in particular in the hemispheres with lesions.

Consideration has therefore been given to improving probabilistic tractography. A technique combining probabilistic tractography with prior anatomical knowledge gave anatomically plausible locations for Meyer's loop, but still required manual region of interest definition (Sherbondy et al., 2008). Complete automation of probabilistic tractography of the optic radiation has been proposed but no measurements of the location of Meyer's loop to confirm its validity were given (Clatworthy et al., 2010) and both these studies only assessed healthy controls.

The aim of this study was therefore to assess a novel method with different aims in comparison to an established method to determine their relative benefits. Visually whilst the core of the tracts was the same, method 1 better delineated the inferior portion of the optic radiation, presumably as the seed region was located within Meyer's loop, which conveys fibres to the inferior optic radiation, whilst method 2 used the LGN as a seed region. Tractography from this region would be expected to better delineate fibres passing directly posteriorly and thus in the more superior part of the optic radiation than those subject to the high curvature of Meyer's loop which was depicted with less consistency and reliability. When planning neurosurgical treatment for epilepsy with a resection in the vicinity of the optic radiation, it is important for a neurosurgeon to be aware of the limitations of the method used when assessing the surgical approach and the risk of causing a visual field deficit. The possibility of either underestimating or overestimating the extent of the tract must always be considered.

The other strengths and weaknesses of the methods fit with the aims of the techniques developed. Method 1 gave reliable visualisation of Meyer's loop with measurements compatible with dissectional data (Ebeling and Reulen, 1988). Using this method, the ML-TP distance has previously been shown to correlate with postoperative visual field outcome and have high reproducibility (correlation coefficient 0.9, average difference 2 mm, maximum 4 mm) (Yogarajah et al., 2009). Method 2 was less consistent in the identification of the Meyer's loop with lower reproducibility of the ML-TP distance particularly in the patient subgroup. In one patient it failed to delineate the anterior part of the optic radiation adjacent to the lesion, as it relied on a seed region and endpoint located either side of a haemosiderin-laden cavernoma causing signal dropout adjacent to the optic radiation. This effect may explain the trend towards ML-TP distance being greater within the patient subgroup using method 2 than method 1 and highlights the importance of assessing tractography techniques both in healthy controls and in the presence of pathology as most studies published to date only include healthy controls. It should however be noted that this reproducibility measure is of a single clinically important measure that method 1 was specifically designed to address. Overall the more posterior part of the tract was far better delineated with method 2 as an endpoint was specified in this area.

Method 2 also required significantly less operator time. The preprocessing of the DTI data common to both methods took 20 min. To process both hemispheres, method 1 required approximately 20–25 min of manual intervention and 60 min of processing time using a single core of a Sun Ultra 27 workstation with a 3.2 GHz Intel Xeon processor. Method 2 required approximately 7 min of manual intervention and 150 min of processing time.

In conclusion the tractography technique thus needs to be chosen according to the question being addressed and the anatomical area of interest. Method 1 would be favoured for anterior temporal lobe resection (patients 2 and 4), whilst method 2 would be favoured for more posteriorly located lesions (patients 1 and 3). By combining these methods and seeding from both the LGN and Meyer's loop the complete optic radiation could be visualised in patients with lesions lying near this structure (Winston et al., 2011).

## Figures and Tables

**Table 1:** Demographic information and imaging diagnoses of the four patients.

Number	Age	Gender	Imaging diagnosis
1	28	F	Right inferior parietal DNET
2	27	M	Left temporal cavernoma
3	42	M	Left occipital DNET
4	18	F	Right fusiform gyrus DNET

**Table 2:** Mean tract volume and ML-TP distance in the different groups (controls, patients) using the two methods. The Dice Similarity Scores for controls and patients are also shown.

	Controls		Patients	
	Method 1	Method 2	Method 1	Method 2
Mean tract volume (voxels)	1455 (220)	1429 (415)	1423 (340)	1003 (263)
ML-TP distance in voxels and	15–18 (16.6)	11–19 (16.9)	14–19 (16.3)	17–26 (18.5)
millimetres (mean)	28–34 (31)	21–36 (32)	26–36 (31)	32–49 (35)
Dice Similarity Score (sd)	0.456 (0.114)		0.318 (0.118)	
